# Systemic inflammatory regulators and age-related macular degeneration: a bidirectional Mendelian randomization study

**DOI:** 10.3389/fgene.2024.1391999

**Published:** 2024-12-13

**Authors:** Xi Liu, Yu Cao, Ying Wang, Lihua Kang, Guowei Zhang, Junfang Zhang, Bai Qin, Ling Yang, Jiawei Luo, Pengfei Li, Wenjing Geng, Min Ji, Huaijin Guan

**Affiliations:** ^1^ Eye Institute, Affiliated Hospital of Nantong University, Medical School of Nantong University, Nantong, Jiangsu, China; ^2^ Department of Medicine, Nantong University, Nantong, Jiangsu, China; ^3^ Department of Ophthalmology, Nantong Third People’s Hospital, Affiliated Nantong Hospital 3 of Nantong University, Nantong, China; ^4^ Department of Ophthalmology, The Affiliated Hospital of Yangzhou University, Yangzhou University, Yangzhou, China

**Keywords:** cytokines, age-related macular degeneration, Mendelian randomization, inflammation, GWAS

## Abstract

**Introduction:**

We investigated the relationship between systematic regulators of inflammation and the risk of age-related macular degeneration (AMD), both wet and dry forms, by using bidirectional, two-sample Mendelian randomization (MR).

**Methods:**

We performed bidirectional two-sample Mendelian randomization analysis using genome-wide study (GWAS) data for 91 plasma proteins from 14,824 individuals of European descent across 11 study groups. Next, we utilized data from the FinnGen consortium to study AMD using the inverse- variance-weighted approach for Mendelian randomization. Additional analyses involved MR-Egger, Weighted median, Weighted mode, MR-PRESSO, and MR- Steiger filtering techniques.

**Results:**

We identified 16 cytokines associated AMD outcomes and post FDR correction, higher levels of fibroblast growth factor 19 and leukemia inhibitory factor receptor were associated with decreased risk for AMD, while higher levels of tumour necrosis factor ligand superfamily member 14 were associated with increased risk for AMD. Additionally, higher levels of interleukin-10 receptor subunit alpha were associated with decreased risk for wet AMD, higher levels of leukemia inhibitory factor receptor were associated with decreased risk for dry AMD, and higher levels of signaling lymphocytic activation molecule were associated with increased risk for dry AMD. Genetic susceptibility to AMD was associated with elevated levels of TNF-related activation-induced cytokines (TNFSF11), and genetic susceptibility to wet AMD was associated with elevated levels of TNFSF11, interleukin-18 receptor 1 (IL18R1), and CUB domain-containing protein 1 (CDCP1).

**Discussion:**

This research enhances our understanding of systemic inflammation in AMD, providing insights into etiology, diagnosis, and treatment of AMD and its forms.

## 1 Background

Age-related macular degeneration (AMD) is a leading cause of vision loss in older individuals because it worsens with time and causes degeneration (Flaxel et al., 2020). The global prevalence of AMD was 8.69% among those aged 45–85 years. It is expected that the patient population would reach 288 million by 2040 (Wong et al., 2014). In recent years, ophthalmology has faced a significant challenge because to the socioeconomic impacts and the increasing prevalence and severity of AMD. The 2010 Global Burden of Disease Study revealed a significant surge of 160% in the number of years individuals lived with disability caused by AMD, emphasizing the immense societal burden associated with vision-related impairments (Vos et al., 2012). AMD can be categorized into neovascular (wet) AMD and non-neovascular (dry AMD). Wet AMD is characterized by the proliferation of predominantly dysfunctional and permeable choroidal arteries that invade the retina. Dry AMD is characterised by the buildup of drusen and the slow progression of geographic atrophy that impacts the retinal pigment epithelium (RPE) and retina (Flaxel et al., 2020). The etiology of AMD remains uncertain, despite the involvement of both hereditary and environmental influences. The complex structure of this disorder is linked to different cellular, metabolic, and molecular processes, with inflammation being a key element in the progression and onset of AMD (Kauppinen et al., 2016).

In AMD, targeting inflammation could potentially offer a promising avenue for intervention (Tan et al., 2020). Neovascular AMD is characterised by the formation of choroidal neovascularization (CNV) due to the activation of inflammatory cytokines, complement system, and regulation of macrophages/microglia (Ishikawa et al., 2016). Anti-vascular endothelial growth factor therapy is primarily used to treat wet AMD, not dry AMD. Administering anti-VEGF drugs via intravitreal injection to patients with wet AMD led to notable changes in cytokines, such as IL-6 and IP-10 (Sato et al., 2018). In the case of dry AMD, the build-up of lipofuscin causes harm to both photoreceptors and RPE cells, thereby interfering with the phagocytic function of lysosomal enzymes. Activated inflammatory cells secrete cytokine to increase the recruitment of inflammatory cells (Damico et al., 2012). Therefore, it can be suggested that inflammation plays distinct roles in the progression of wet and dry forms of AMD. The available evidence indicates a clear correlation between inflammation and AMD, including its subtypes, and that inflammation functions as both the catalyst and the consequence of a detrimental cycle. Researchers at the First Ophthalmic Clinic, Medical University of Pomerania in Szczecin, Poland, recruited 179 patients with wet AMD, 175 patients with dry AMD, and 121 control people for a study. Plasma samples were collected and analyzed using Luminex technology to assess levels of soluble inflammatory factors. Elevated levels of IL-6, GM-CSF, and IFN-γ were found in wet AMD, but reduced levels of IL-1β, IL-5, IL-10, and IL-12 were seen. Dry AMD was identified as an independent variable linked to elevated levels of GM-CSF and IL-6, and decreased levels of TNF-, IL-1, IL-2, IL-5, IL-10, and IL-12, as determined by multivariate analysis (Litwińska et al., 2019). At present, there is insufficient data from clinical studies conducted on a population level to establish a direct cause-and-effect connection between inflammation and AMD. By employing the Mendelian Randomization (MR) methodology in our research, we investigated the association between systematic regulators of inflammation and AMD. As a result, our research results are less prone to reverse causation and potential confounding factors. Additionally, we employed the most extensive genome-wide association study (GWAS) dataset available to identify single nucleotide polymorphisms (SNPs) associated with systemic inflammatory regulators and AMD.

Mendelian randomization (MR) circumvents biases by employing genetic variants to establish causal relationships; it is analogous to a large-scale randomised controlled trial in nature (Lawlor et al., 2008a). The potential therapeutic utility of inflammatory modulators in AMD has been illuminated in a recent MR analysis, which established a causal link between increased levels of C-reactive protein and the disease AMD (Han et al., 2020). Bidirectional MR analysis, an extension of traditional MR, has been crucial in revealing intricate relationships within biological systems, like feedback loops linking exposure and outcome variables (Li et al., 2023).

We conducted a comprehensive investigation of the potential link between AMD and systemic inflammatory regulators using bidirectional MR analysis. The analysis encompassed not only AMD but also the distinction between wet and dry AMD, taking into account the various clinical subtypes of the disease.

## 2 Methods

### 2.1 Study design

**FIGURE 1:**
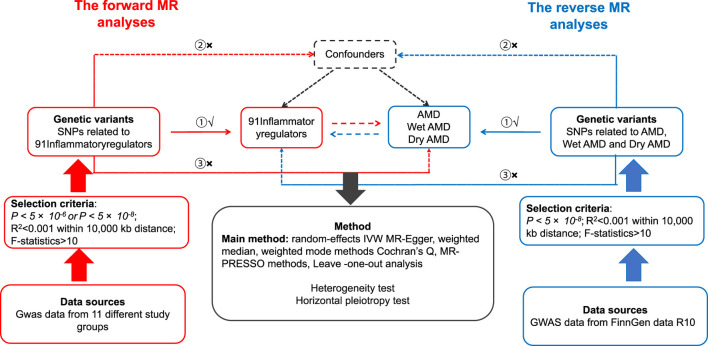
Hypotheses of a study using Mendelian randomization to examine the potential link between inflammatory regulators and the likelihood of developing AMD. ① Relevance Assumption, ② Independence Assumption, ③ Exclusion Restriction Assumption. SNPs: genetic variations at the level of individual nucleotides.

The Mendelian randomization method used genetic variants as instrumental variables (IVs) to determine a causal relationship between 91 inflammatory cytokines and AMD (Lawlor et al., 2008b; Widding-Havneraas and Zachrisson, 2022). In this investigation, valid IVs must satisfy the following three conditions (Smith and Ebrahim, 2003; Davey Smith and Hemani, 2014): (i) SNPs exhibit a robust correlation with the exposure; (ii) SNPs keep their association with the outcome unaffected by confounding variables; and (iii) Exposure is the only factor that connects SNPs to the outcome (Choi et al., 2019). The diagram illustrating the study’s flowchart can be found in Figure 1. Analysing the correlation between 91 inflammatory regulators and AMD, this research utilised deidentified public summary-level data that is available for free distribution. All of the GWASs utilised in this investigation received approval from the ethics committees of their respective institutions.

### 2.2 Data source for inflammatory regulators

We gathered genetic data from 11 different study groups, analyzing the levels of 91 inflammatory markers in the bloodstream (Supplementary Table S1). The study involved a cohort of 14,824 individuals who had both their genetic information analyzed using genome-wide techniques and their plasma proteomic data measured using the Olink Target Inflammation panel (Zhao et al., 2023). The cytokine source information has been appended to the Supplementary Table S2.

### 2.3 Data source for AMD

FinnGen is a joint project including both public and private entities. It merges electronic health records from Finnish health registries with imputed genetic data obtained from newly acquired and existing samples housed in Finnish biobanks (Kurki et al., 2023). Its primary objective is to offer novel perspectives on the genetics of diseases (https://www.finngen.fi/en). FinnGen is a joint effort that involves nine Finnish biobanks, research institutions, universities, and university hospitals, as well as thirteen international pharmaceutical industry partners and the Finnish Biobank Cooperative (FINBB). We downloaded the available summary statistics from the public release of FinnGen data R10 results for AMD (9,721 cases and 381,339 controls), wet AMD (5,239 cases and 273,920 controls) and dry AMD (6,651 cases and 272,504 controls). The International Classification of Diseases, Ninth Revision (ICD-9; 3625A and 3625B) and ICD-10 (H35.30) supply the definition of AMD as an endpoint. The entire membership of the FinnGen consortium consisted of Finnish subjects.

### 2.4 Selection of genetic instrumental variables

To fulfill the MR assumptions, all SNPs were selected based on their independent and strong associations with exposures as reported in the GWAS at a significance level of *P* < 5 × 10^−8^, as determined by R^2^ < 0.001 within 10 Mb. The number of systemic inflammatory regulators with three or more independent SNPs that reached genomewide significance was 62, and we opted for a more lenient threshold of 5 × 10^−6^ for the remaining 29 inflammatory factors in order to identify additional SNPs associated with these regulators (Chen et al., 2022) (Supplementary Table S3). As previously described, the thresholds are suitable for selecting genetic instrumental variables (Burgess et al., 2013).

To identify independent SNPs for use as IVs, a rigorous aggregation procedure was implemented. This involved ensuring that the linkage disequilibrium coefficient r^2^ between SNPs was below 0.001 within a window of 10,000-kb, with reference to the European 1,000G panel. In cases where the intended instrumental SNPs were unavailable, proxy SNPs were utilised, with an r-value greater than or equal to 0.8. To assess the efficacy of the chosen genetic predictors for regulating inflammation in the body, we computed an F statistic for each specific nucleotide variation by employing the formula (β/σ)^2^ (where β denotes SNP-exposure association and σ represents variance) (Bowden et al., 2016a). In general, when F is greater than 10, feeble IVs do not contribute to any discernible bias. The R^2^ value was employed to determine the extent to which instruments could explain the variability in exposures. For this study, only exposures with three or more valid instrumental variables were included for analysis.

To explore the possible impact of AMD on systemic inflammatory regulators, we employed a genome-wide significance threshold (*P* = 5 × 10^−8^) for our analysis. The remaining selection procedures for systemic inflammatory regulators were identical. The SNPs that were chosen are listed in Supplementary Table S4.

### 2.5 MR analysis

The standard inverse‐variance weighted (IVW) method was utilized in the bidirectional two-sample MR analysis to estimate causal effects while maintaining effect consistency. IVW generates a consistent estimation of the causal relationships between exposures and outcomes by applying inverse variance weighting to the associations between SNPs and outcomes. This method is highly effective, relying on the assumption that each selected SNP is valid and there is no presence of horizontal pleiotropy. Conversely, if IVs fail to adhere to the assumption of “no horizontal pleiotropy,” the estimated results obtained through IVW will be significantly distorted (Burgess et al., 2013). Complementary analyses were conducted utilising the weighted median approach (Bowden et al., 2016b), MR-Egger regression (Bowden et al., 2015), and weighted mode method (Hartwig et al., 2017). Consistent estimations are guaranteed by the weighted median method, provided that a minimum of 50% of the weights utilised in the analysis are derived from valid instrumental variables. While the MR-Egger regression may address directional pleiotropy, its effectiveness is limited. In contrast, the weighted mode approach maintains consistency by relying on a larger number of valid instruments to derive comparable individual-instrument causal effect estimates. The existence of pleiotropy was evaluated by employing both the MR-Egger test’s intercept and the MR-PRESSO Global test. A significance level of less than 0.05 suggested that the exposure may not be the sole mechanism by which IVs could impact the outcome. Furthermore, the variability of IVs was evaluated by employing Cochrane’s Q statistic; a *p*-value below 0.05 was considered as suggestive of heterogeneity (Hemani et al., 2018). The statistical significance of the MR effect estimates was determined by using a false discovery rate (FDR) threshold of less than 0.05 in order to account for multiple testing (Liu et al., 2017). The MR studies were conducted in R (version 4.0.0) using the R packages “TwosampleMR” and “MR-PRESSO”. Furthermore, funnel plots and scatter plots were employed. The scatter plots illustrated that the results remained unaltered by any exceptional data points.

## 3 Results

### 3.1 Choice of IVs

In our research, we conducted an initial screening of 91 inflammatory factors to identify IVs. This process yielded a cumulative count of 731 SNPs that satisfied our pre-established screening criteria. It is worth noting that all these SNPs exhibited significant association strength, with F-statistics ranging from 20.85 to 1,477.14. We examined 29 IVs associated with AMD, 23 IVs linked to wet AMD, and 21 IVs associated with dry AMD in the reverse MR analysis. All these SNPs demonstrated significant association strength, with F-statistics ranging from 30.17 to 1,061.2.

### 3.2 Relationship of causation between inflammatory factors and AMD

**FIGURE 2:**
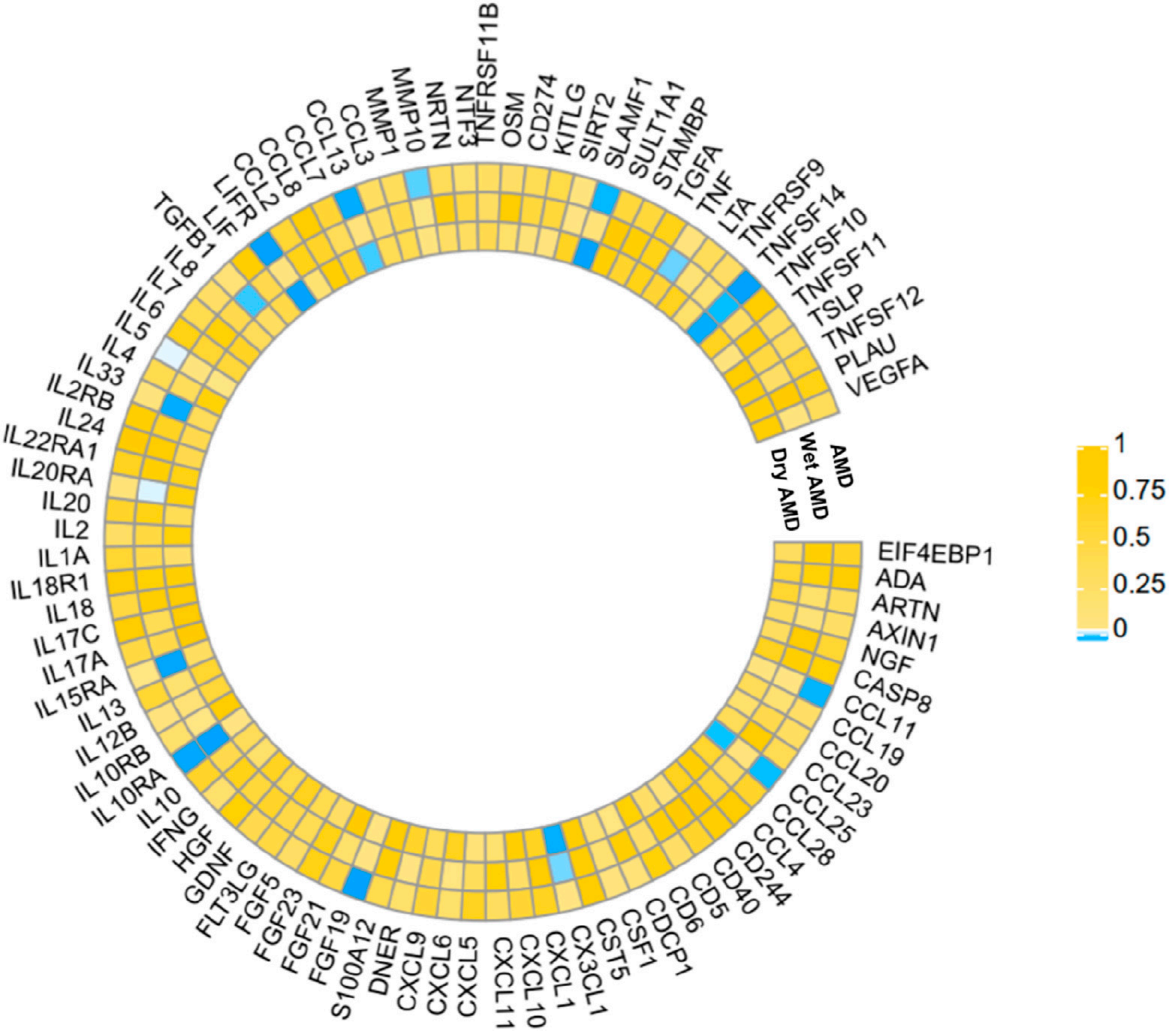
The statistical significance of the relationships between 91 systemic inflammatory regulators and AMD diseases is presented. The *p* values obtained from the fixed-effects IVW method are displayed. The abbreviations are provided in the Supplementary material.

**FIGURE 3:**
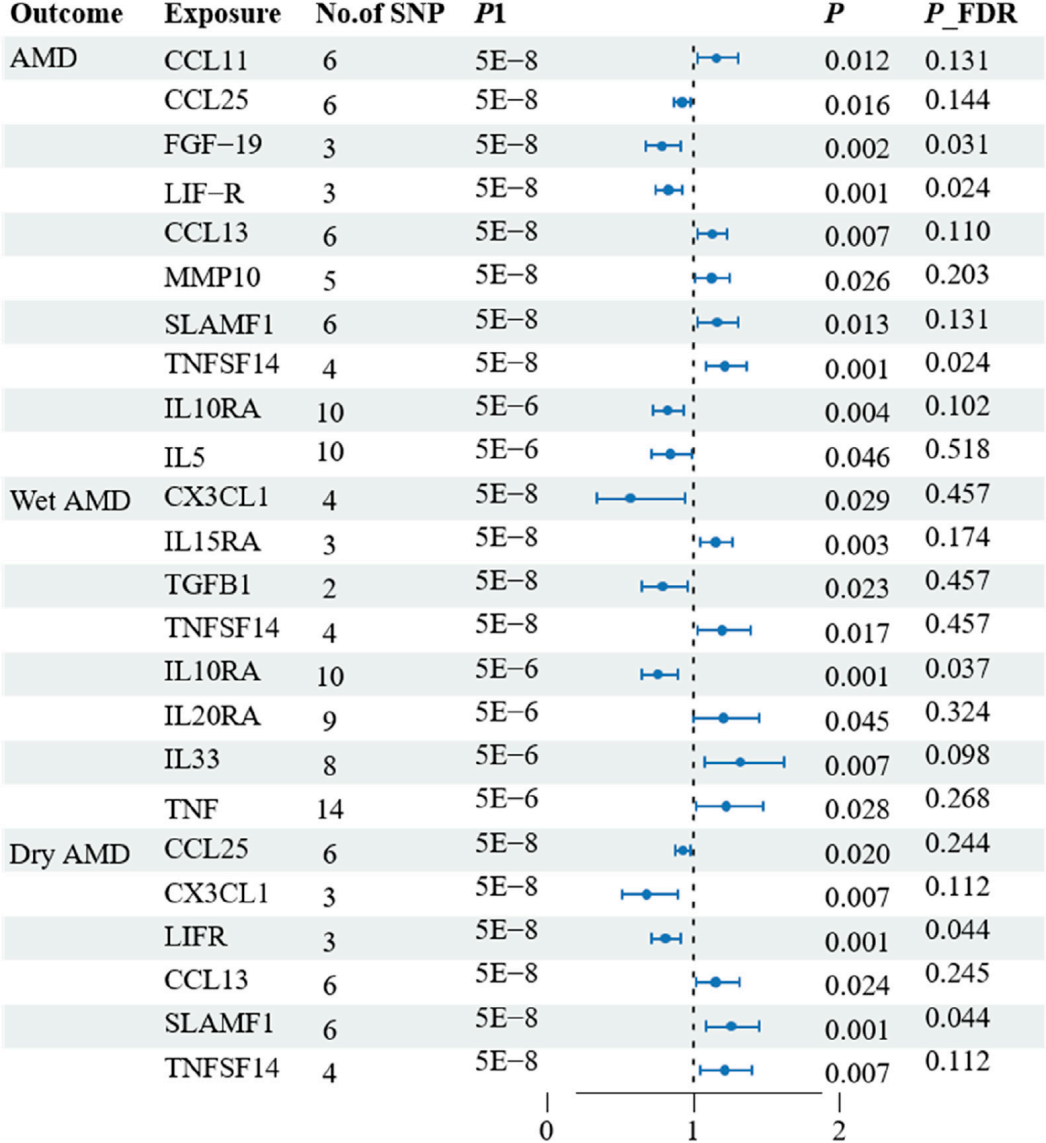
Associations of systemic inflammatory regulators with the AMD including wet AMD and dry AMD. The presented results display associations with a significance level of *p* < 0.05 in the IVW models. * Subsequent analysis was conducted to reassess the causal impacts of the exposures on the outcomes by excluding outliers identified through MR-PRESSO test.

**FIGURE 4:**
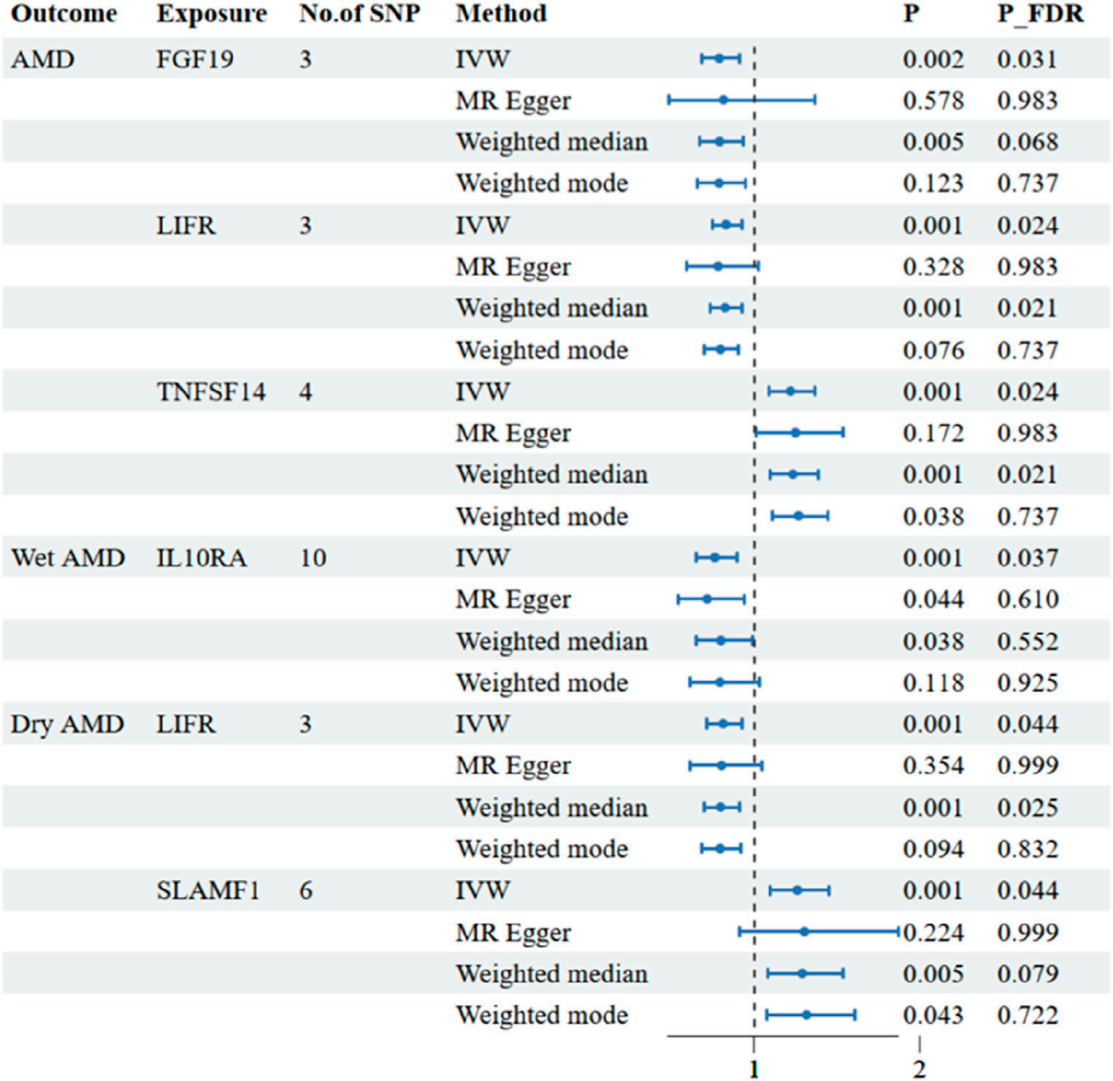
Associations of systemic inflammatory regulators with the AMD including wet AMD and dry AMD. The presented results display associations with a significance level of *p* < 0.05 in the IVW models after FDR correction. MR, Mendelian randomization; SNP, single nucleotide polymorphism; IVW: inverse variance weighted.

**FIGURE 5:**
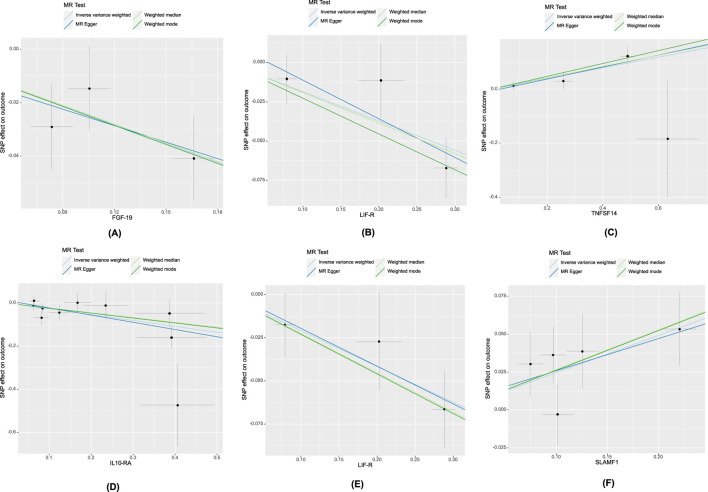
Scatter plot of systemic inflammatory regulators-associated SNPs with risk of AMD. The genetic relationship between FGF-19, LIFR, TNFSF14 and AMD, denoted as **(A–C)**, respectively. **(D)**: The genetic relationship between IL10-RA and wet AMD. The genetic relationship between LIF-R, SLAMF1 and dry AMD, denoted as **(E, F)**, respectively. Abbreviations: FGF-19, Fibroblast growth factor 19; LIF-R, Leukemia inhibitory factor receptor; TNFSF14, Tumor necrosis factor ligand superfamily member 14; IL10-RA, Interleukin-10 receptor subunit alpha; LIFR, Leukemia inhibitory factor receptor; SLAMF1, Signaling lymphocytic activation molecule.

The research has shown a causal relationship between changes in AMD and 10 inflammatory variables (Figure 2). The IVW genetic prediction method revealed the presence of elevated levels of Eotaxin (CCL11) (OR = 1.164, 95% CI 1.034–1.311, *P* = 0.012), Monocyte chemoattractant protein-4 (CCL13) (OR = 1.131, 95% CI 1.034–1.238, *P* = 0.007), Matrix metalloproteinase-10 (MMP-10) (OR = 1.129, 95% CI 1.015–1.257, *P* = 0.026), Signaling lymphocytic activation molecule (SLAMF1) (OR =, 95% CI 1.015–1.257, *P* = 0.026) and Tumor necrosis factor ligand superfamily member 14 (TNFSF14) (OR = 1.219, 95% CI 1.086–1.369, *P* = 0.001) were associated with an increased risk of dry AMD. As indicated by the IVW method for genetic prediction, increased concentrations of C-C motif chemokine 25 (CCL25) (OR = 0.932, 95% CI 0.879–0.989, *P* = 0.016), Fibroblast growth factor 19 (FGF19) (OR = 0.790, 95% CI 0.683–0.914, *P* = 0.002) and Leukemia inhibitory factor receptor (LIFR) (OR = 0.830, 95% CI 0.745–0.925, *P* = 0.001) were associated with a decreased risk of AMD (Figure 3). The aforementioned findings exhibited similarity to the outcomes derived from MR-Egger, weighted median, and weighted modal analyses. The correlation between genetically predicted inflammatory regulators and AMD is depicted in Figure 5 as a scatter diagram. The absence of horizontal pleiotropy was confirmed by the MR-Egger and MR-PRESSO analyses (*P* > 0.05). Furthermore, no variants that had a substantial impact on the overall outcome were identified by the leave-one-out test (Supplementary Figure S1). As determined by the FDR correction test, elevated moist AMD remained strongly causally related to lower levels of FGF19 and LIFR and higher levels of TNFSF14 (Figure 4).

### 3.3 Relationship of causation between inflammatory factors and wet AMD

The research discovered signs of a cause-and-effect connection between 8 inflammatory markers and a higher likelihood of developing wet AMD (Figure 2). The IVW genetic prediction method revealed the presence of elevated levels of Interleukin-15 receptor subunit alpha (IL15RA) (OR = 1.158, 95% CI 1.052–1.275, *P* = 0.003), Tumor necrosis factor ligand superfamily member 14 (TNFSF14) (OR = 1.202, 95% CI 1.033–1.397, *P* = 0.017), Interleukin-20 receptor subunit alpha levels (IL20RA) (OR = 1.209, 95% CI 1.004–1.456, *P*-FDR = 0.045), Interleukin-33 levels (IL33) (OR = 1.324, 95% CI 1.081–1.622, *P* = 0.007) and Tumor necrosis factor levels (TNF) (OR = 1.230, 95% CI 1.023–1.478, *P* = 0.028) were associated with an increased risk of AMD. The IVW method for genetic prediction revealed that higher levels of fractalkine levels (CX3CL1) (OR = 0.572, 95% CI 0.346–0.946, *P* = 0.029), latency-associated peptide transforming growth factor beta 1 (TGFB1) (OR = , 95% CI 0.650–0.968, *P* = 0.023) and Interleukin-10 receptor subunit alpha (IL10RA) (OR = 0.762, 95% CI 0.646–0.899, *P* = 0.001) were associated with a decreased risk of AMD (Figure 3). No indications of heterogeneity were detected during the examination of inflammatory factors. Both the MR-Egger and MR-PRESSO assays showed no evidence of horizontal pleiotropy (*P* > 0.05). The relationship between genetically predicted regulators of inflammation and wet AMD is illustrated in Figure 5 using a scatter plot. The leave-one-out analysis did not reveal any differences that had a substantial impact on the overall results (Supplementary Figure S2). As determined by the FDR correction test, elevated moist AMD remained strongly causally related to lower levels of IL10RA (Figure 4).

### 3.4 Relationship of causation between inflammatory factors and dry AMD

The findings indicate a causal link between alterations in dry AMD and six inflammatory factors (Figure 2). The IVW approach employed for genetic prediction demonstrated an association between elevated concentrations of monocyte chemoattractant protein-4 (CCL13) (OR = 1.159, 95% CI 1.020–1.317, *P* = 0.024), signaling lymphocytic activation molecule (SLAMF1) (OR = 1.261, 95% CI 1.094–1.453, *P* = 0.001) and tumor necrosis factor ligand superfamily member 14 (TNFSF14) (OR = 1.218, 95% CI 1.056–1.406, *P* = 0.073) were associated with an increased risk of dry AMD. The IVW genetic prediction method indicated a rise in the levels of C-C motif chemokine 25 (CCL25) (OR = 0.932, 95% CI 0.879–0.989, *P* = 0.020), CX3CL1 (OR = 0.684, 95% CI 0.519–0.903, *P* = 0.007) and LIFR (OR = 0.812, 95% CI 0.715–0.923, *P* = 0.001) were associated with a decreased risk of dry AMD (Figure 3). No indications of heterogeneity were detected during the course of our investigation. The corresponding scatter diagrams depicting the associations between these inflammatory regulators and dry AMD are presented in Figure 5. The absence of horizontal pleiotropy was not supported by the results of the MR-Egger and MR-PRESSO assays (*P* > 0.05). No associated variants that had a substantial impact on the overall findings were identified by the leave-one-out test (Supplementary Figure S3). Increased SLAMF1 levels and decreased LIFR levels were found to have a significant causal connection with a higher risk of dry AMD, as indicated by the FDR correction test (Figure 4).

### 3.5 Reverse analysis

**FIGURE 6:**
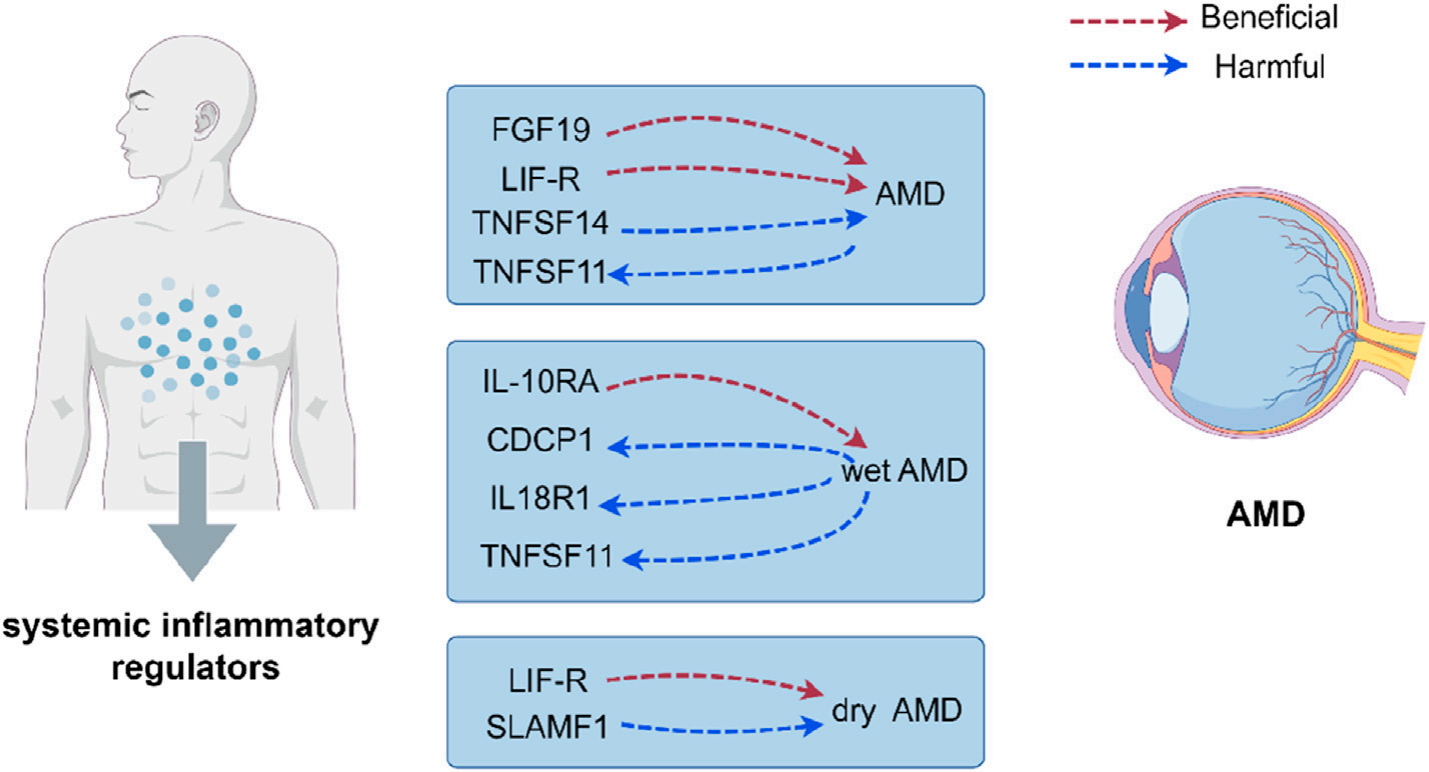
Reciprocal relationship between systemic inflammatory markers and the development of AMD.

This study identified causal correlations between 91 inflammatory factors and outcomes of AMD, including wet AMD and dry AMD, using forward analyses. In order to ascertain the genetic correlation between AMD and 91 inflammatory factors, we also performed an inverted study. Following FDR correction, the IVW analysis indicated that AMD could result in elevated TNFSF11 (beta = 0.067, 95% CI 0.036–0.097, *P* < 0.001) and wet AMD may lead to higher levels of CDCP1 (beta = 0.057, 95% CI 0.026–0.087, *P* < 0.001), IL18R1 (beta = 0.055, 95% CI 0.024–0.085, *P* < 0.001) and TNFSF11 (beta = 0.054, 95% CI 0.023–0.086, *P* = 0.001). However, we did not find any significant correlations between dry AMD and other inflammatory factors. Figure 6 illustrates a reciprocal cause-and-effect connection between inflammatory variables and AMD. Our investigation yielded no discernible signs of horizontal pleiotropy (*P* > 0.05).

## 4 Discussion

Our research represents the initial comprehensive and extensive MR analysis conducted thus far, focusing on exploring the genetic causality between systemic inflammatory regulators and AMD, encompassing both wet AMD and dry AMD. Prior research has predominantly concentrated at the level of cells or animals, investigating localised inflammation within ocular tissues or cells as opposed to the organism’s systemic inflammatory response (Tan et al., 2020). In clinical environments, observational studies frequently encounter constraints such as the presence of confounding variables and bias due to reverse causation. These limitations can potentially distort the causal connections between different variables. Through the integration of data obtained from large-scale population-based GWAS, our research has effectively identified five genetically associated inflammatory factors that are implicated in various AMD-related outcomes. We observed robust causal associations between FGF19, LIFR, TNFSF14 and AMD, IL10RA and wet AMD, as well as LIFR, SLAMF1and dry AMD. A subsequent analysis unveiled a correlation between genetic susceptibility to AMD and an increase in TNFSF11 expression, while genetic susceptibility to wet AMD was linked to increased expression of CDCP1, IL18R1, and TNFSF11. These results emphasize the influence of genetic mechanisms on the control of systemic inflammatory factors in diverse cases of AMD.

Inflammation is a complex physiological response that is initiated in response to tissue damage or the presence of foreign substances. It provides substantial short-term advantages by eliminating detrimental stimuli and commencing the tissue restoration process (Megha et al., 2021). However, detrimental consequences have been associated with the prolonged presence of chronic inflammation. There is evidence to suggest that persistent moderate inflammation can contribute to the development of chronic diseases, such as cancer, diabetes, and neurological disorders, among others. While the exact cause of AMD is affected by various factors, there is strong evidence indicating that inflammation plays a crucial role in its development. RPE cells secrete a substantial amount of inflammatory mediators, actively contributing to the initiation and progression of an inflammatory cascade. AMD is caused by an extended duration of imbalanced pro-inflammatory and anti-inflammatory reactions. The involvement of pro-inflammatory cytokines IL-1β, IL-6, IL-8, IL-12, IL-17, CSF-1, TNF-α, IFN-β, and IFN-γ in addition to anti-inflammatory cytokines IL-4, IL-10, and TGF-β is significant in the formation of CNV through diverse signaling pathways (Tan et al., 2020). The fibrotic process involves pro-inflammatory cytokines such as IL-2 and IL-6, as well as the anti-inflammatory cytokine IL-10 and TGF-beta. Furthermore, the recruitment of diverse inflammatory cell types—including innate immune cells such as macrophages, dendritic cells, and neutrophils—as well as adaptive immune cells such as T lymphocytes and B lymphocytes—into the eye is intricately linked to the inflammatory response. Immune cells possess both the ability to secrete cytokines themselves and are also influenced by them. Increasing evidence substantiates the notion that inflammation contributes to AMD. Although several systemic and local inflammatory molecules, including CRP, NLR, and active monocytes (Kauppinen et al., 2016), have been suggested as potential biomarkers for AMD, no particular and trustworthy markers have been discovered to date.

A set of five systemic inflammatory regulators was identified, the concentrations of which were correlated with AMD disorders based on genetic analysis. To begin with, it was necessary to elucidate the inverse relationship between FGF-19, LIF-R, and AMD. Firstly, Fibroblast growth factor signaling is of utmost importance in the formation and progression of various tissues, including ocular tissue (Fuhrmann, 2010). The results of *in-vitro* studies demonstrate that FGF-19 exerts neuroprotective effects on photoreceptors in adult mammals (Siffroi-Fernandez et al., 2008). Furthermore, the leukemia inhibitory factor receptor (LIF-R), a member of the type 1 cytokine receptor family weighing approximately 190 kDa, facilitates intracellular signaling pathways that enhance cell viability in nerve cells and trigger an anti-inflammatory state in T cells and macrophages (Gadina et al., 2001). The dual properties of LIFR signaling make it a promising therapeutic target for stroke and other neurological injuries. Studies on animals have revealed the significant contribution of LIFR in innate protective mechanisms within the retina, resulting in the preservation of photoreceptors (Davis and Pennypacker, 2018). The results of our study indicate that LIF-R exerts a beneficial effect in the context of dry AMD. Consistent with our expectations, we have identified FGF-19 and LIF-R as potential protective factors against AMD. However, additional extensive examinations are necessary to authenticate this correlation and clarify the underlying mechanistic pathways. What’s more, inflammatory factors TNFSF14 were positively associated with MM. TNFSF14, alternatively referred to as LIGHT, is a protein categorized under the TNF superfamily. Its primary function involves regulating immune cell activity and modulating T lymphocyte functions. Furthermore, it is involved in the development of persistent inflammation, autoimmune disorders, and specific malignancies. Studies conducted on animals have provided evidence for the crucial role of LIGHT signaling in different autoimmune disorders, including inflammatory bowel disease, nephritis, diabetes, and arthritis (Wang et al., 2005; Ware, 2005). Clinical observations have demonstrated that elevated levels of TNFSF14 may serve as an indicator of prospective clinical consequences in patients who have been diagnosed with stable coronary artery disease (Hsu et al., 2019). Our MR analysis provides novel evidence suggesting an association between TNFSF14 and AMD.

Wet AMD was found to have a significant inverse correlation with IL-10RA expression, according to the results of our study. Secreted by a variety of immune cells, including T helper cells, macrophages, monocytes, and B cells, IL-10 is a soluble protein. It exhibits an extensive array of immunosuppressive and immunostimulatory characteristics. IL-10 has the ability to prevent programmed cell death in B lymphocytes, hence promoting their differentiation, proliferation, and MHC class II molecule synthesis. Moreover, it enhances the cytotoxic functions of natural killer cells. While IL-10 protects T cells from undergoing apoptotic cell death, it inhibits the survival of developing mast cells and macrophages by inducing mitochondrial apoptosis and blocking the function of growth factor receptors. This naturally occurring cytokine can be found in humans, mice, and other organisms. Particularly in ocular inflammations, IL-10 functions as a pivotal anti-inflammatory agent exhibiting formidable anti-angiogenic properties (Mosmann, 1994). Unexpectedly, IL-10 exhibits captivating antiangiogenic properties in various ocular regions, including the conjunctiva, cornea, uvea, retina, and orbit (Ghasemi et al., 2012). The fragility and hemorrhage of intraocular neovascularization can result in ocular dysfunction and eventual vision loss, with wet AMD being the most prevalent condition. The intricate regulation of neovascularization within the eye involves a dynamic interaction between multiple factors, such as angiogenic agents, inhibitory substances, and adhesive molecules. Among these components, certain ones have been recognized as promising therapeutic targets for addressing this condition (Yoshida et al., 1999). The research findings revealed a reduction in plasma concentrations of IL-10RA in patients diagnosed with wet AMD, suggesting that this protein may serve as a viable target for forthcoming therapeutic approaches targeting this disorder.

Dry AMD is a multifactorial disease of intricate nature, with an incompletely comprehended pathogenesis. Existing evidence indicates that the involvement of inflammation contributes to its progression (Anderson et al., 2002). The existence of drusen is regarded as an initial signifier for the presence of dry AMD, which can progress to form drusen-like RPE detachment through expansion and merging (Ebrahimi and Handa, 2011). Drusen contain various proinflammatory factors, including components of the complement pathway and lipofuscin-related products, making them significant contributors to the progression of dry AMD (Gehrs et al., 2010; de Guimaraes et al., 2022). SLAMF1, also known as CD150, is a 70 kDa protein belonging to the immunoglobulin superfamily. It exists in two distinct forms: one that is bound to a membrane and the other of which is soluble (Cocks et al., 1995). SLAMF1 acts as a co-stimulatory receptor that colocalizes with CD3 and regulates downstream signaling during T cell activation (Howie et al., 2002). Beforehand investigations have established a correlation between this specific protein and autoimmune disorders, as individuals diagnosed with rheumatoid arthritis and systemic lupus erythematosus have exhibited heightened expression of SLAMF1 on lymphocytes located both peripherally and within the body (Karampetsou et al., 2017). Our research is the initial to propose increased levels of peripheral SLAMF1 in individuals diagnosed with dry AMD. The observational results provide backing to the theory that retinal inflammation has a substantial impact on altering dry AMD. Moreover, inhibiting inflammatory pathways is expected to slow down visual loss in these patients, thereby greatly improving their quality of life. Future research will focus on elucidating the cellular mechanisms through which inhibition of inflammation delays retinal degeneration.

The simultaneous findings indicate that AMD may also exert an impact on certain inflammatory regulators. Notably, robust associations were observed between AMD and TNFSF11, as well as wet AMD and CDCP1, IL18R1, TNFSF11. The association of certain cytokines with psychiatric disorders has been previously documented. Recent research indicates that cytokines might have a significant impact on the progression of watery AMD. Multiple research studies have indicated that wet AMD is characterized by an ongoing, mild inflammatory process, where various aqueous and vitreous cytokines contribute to the disease’s progression. The aforementioned include tissue factor, VEGF, platelet-derived growth factor, and tissue inhibitor of metalloproteinases (Agrawal et al., 2019; Minaker et al., 2021). Considering the association between other cytokines and anatomical as well as functional therapeutic responses, it may be imperative to target multiple factors beyond VEGF for achieving long-term stability. Rapid progress in analytical techniques has facilitated an expansion of research investigating the concentrations of intraocular cytokines in individuals afflicted with moist AMD. The objective of these research endeavours is to contribute to the identification of prospective biomarkers that may be the focus of future therapeutic initiatives. Additionally, prognostic biomarkers under consideration may hold significant value in predicting patients’ clinical progression and response to treatment. Despite this, it is essential to recognise that observational studies are susceptible to a variety of confounding variables, which can complicate the establishment of causal relationships. By solely focusing on the concentrations of genetically determined inflammatory regulators, our findings suggest a possible protective mechanism within the body against inflammation and further retinal degeneration in individuals with AMD disease. Consequently, these inflammatory regulators hold promise as potential diagnostic biomarkers; nevertheless, additional research is necessary to validate their actual clinical utility. The association between AMD and CDCP1, IL18R1, TNFSF11 represents a novel finding; however, limited relevant studies impede our understanding of these connections and warrant further exploration through population-based observations and experimental investigations.

Our study’s robustness is derived from the utilisation of genetic instrumental variables, which enabled us to conduct a comprehensive MR analysis investigating the association between inflammatory factors and the probability of developing AMD and its subcategories. This pioneering endeavor holds significant implications for elucidating whether genetic predisposition to AMD induces alterations in circulating inflammatory factors, as well as determining the impact of high or low levels of these factors on AMD risk, aspects that have not been explored in previous MR studies. This study has certain limitations. Firstly, a valid and robust IV is essential for obtaining reliable results in MR analysis. Although neither the MR-PRESSO test nor the MR-Egger intercept detected substantial pleiotropy or confounding factors, it is critical to recognise that MR analysis makes it difficult to completely rule out this possibility. Additionally, a larger sample size would enhance the precision of assessing genetic effects on exposure due to the relatively small proportion of variance explaining their association. However, F-statistics suggest that this limitation does not significantly affect our findings. Thirdly, considering the divergent results observed for associations between blood and eye regulators of inflammation, it is possible that intraocular inflammation may have distinct effects requiring further investigation; unfortunately, limited availability of intraocular inflammation data has impeded progress in this research area. Furthermore, it is important to acknowledge that our findings are predominantly based on data collected from European populations. Consequently, it is imperative to replicate these analyses using extensive GWAS data when it becomes accessible for diverse populations.

## 5 Conclusion

Our bidirectional MR study suggests that a group of systemic regulators linked to inflammation may either contribute to the advancement of AMD or be affected by AMD and its subtypes. While additional research is required to elucidate the precise mechanisms that connect regulators of systemic inflammation and AMD, this study offers supplementary perspectives on the association between systemic inflammation and the disease. These findings have the potential to offer new clues regarding the causes of this disease as well as biomarkers for clinical diagnosis and treatment.

## Data Availability

The original contributions presented in the study are included in the article/Supplementary Material, further inquiries can be directed to the corresponding authors.
