# Supplementary material for: Systemic inflammatory regulators and age-related macular degeneration: a bidirectional Mendelian randomization study
Source: Front Genet. 2024 Dec 13;15:1391999. doi: 10.3389/fgene.2024.1391999 (PMC11671502; doi:10.3389/fgene.2024.1391999)
Supplement: Supplementary file 1 [file Table2.docx]

**Supplementary Content**

**Supplementary Figure 1**. MR leave-one-out sensitivity analysis of systemic inflammatory regulators-associated SNPs with risk of AMD.

**Supplementary Figure 2**. MR leave-one-out sensitivity analysis of systemic inflammatory regulators-associated SNPs with wet AMD.

**Supplementary Figure 3**. MR leave-one-out sensitivity analysis of systemic inflammatory regulators-associated SNPs with dry AMD.

**
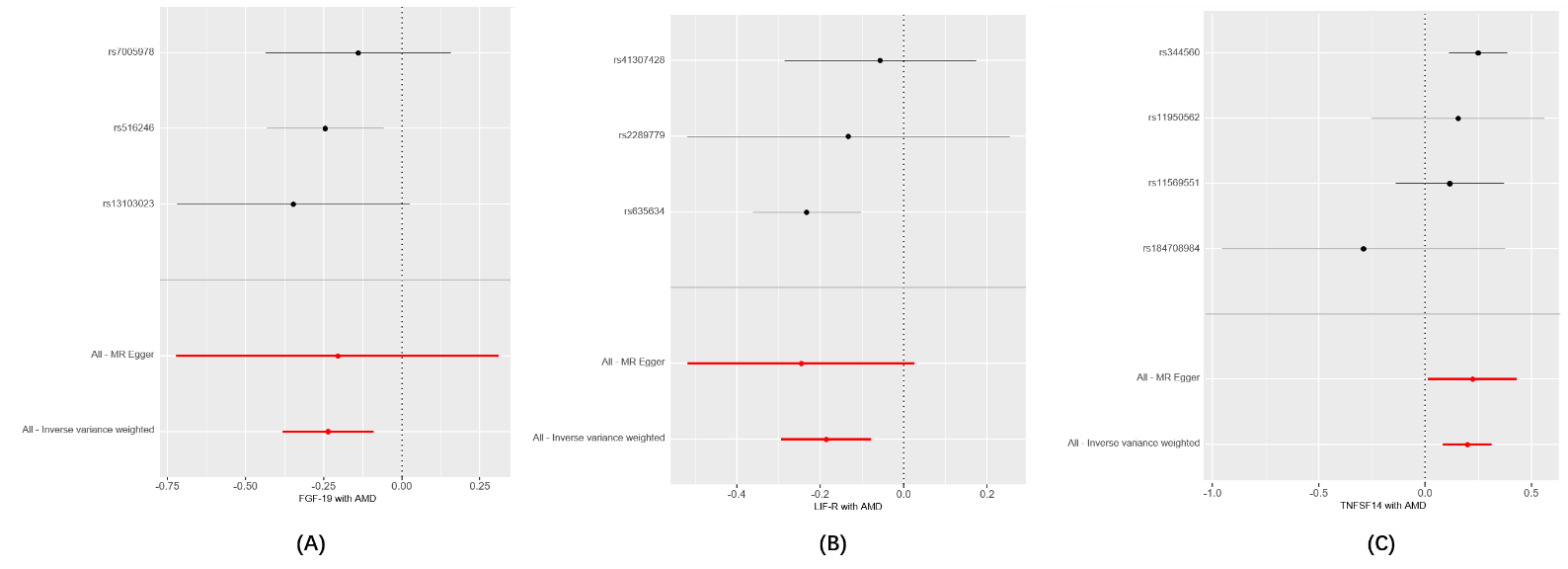
**

**Supplementary Figure 1** MR leave-one-out sensitivity analysis of systemic inflammatory regulators-associated SNPs with risk of AMD. The genetic relationship between FGF-19, LIFR, TNFSF14 and chronic kidney disease, denoted as A to C, respectively. Abbreviations: FGF-19, Fibroblast growth factor 19; LIF-R, Leukemia inhibitory factor receptor; TNFSF14, Tumor necrosis factor ligand superfamily member 14.

**
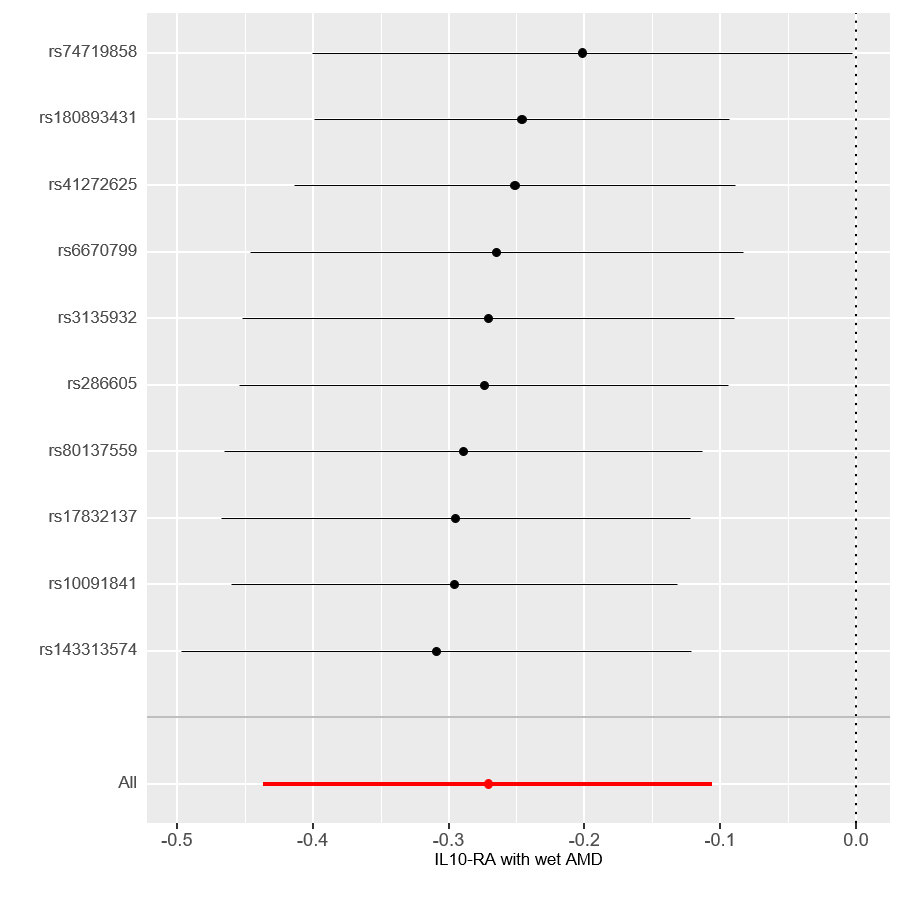
**

**Supplementary Figure 2**. MR leave-one-out sensitivity analysis of systemic inflammatory regulators-associated SNPs with risk of wet AMD. The genetic relationship between IL10-RA and AMD. Abbreviations: IL10-RA, Interleukin-10 receptor subunit alpha.


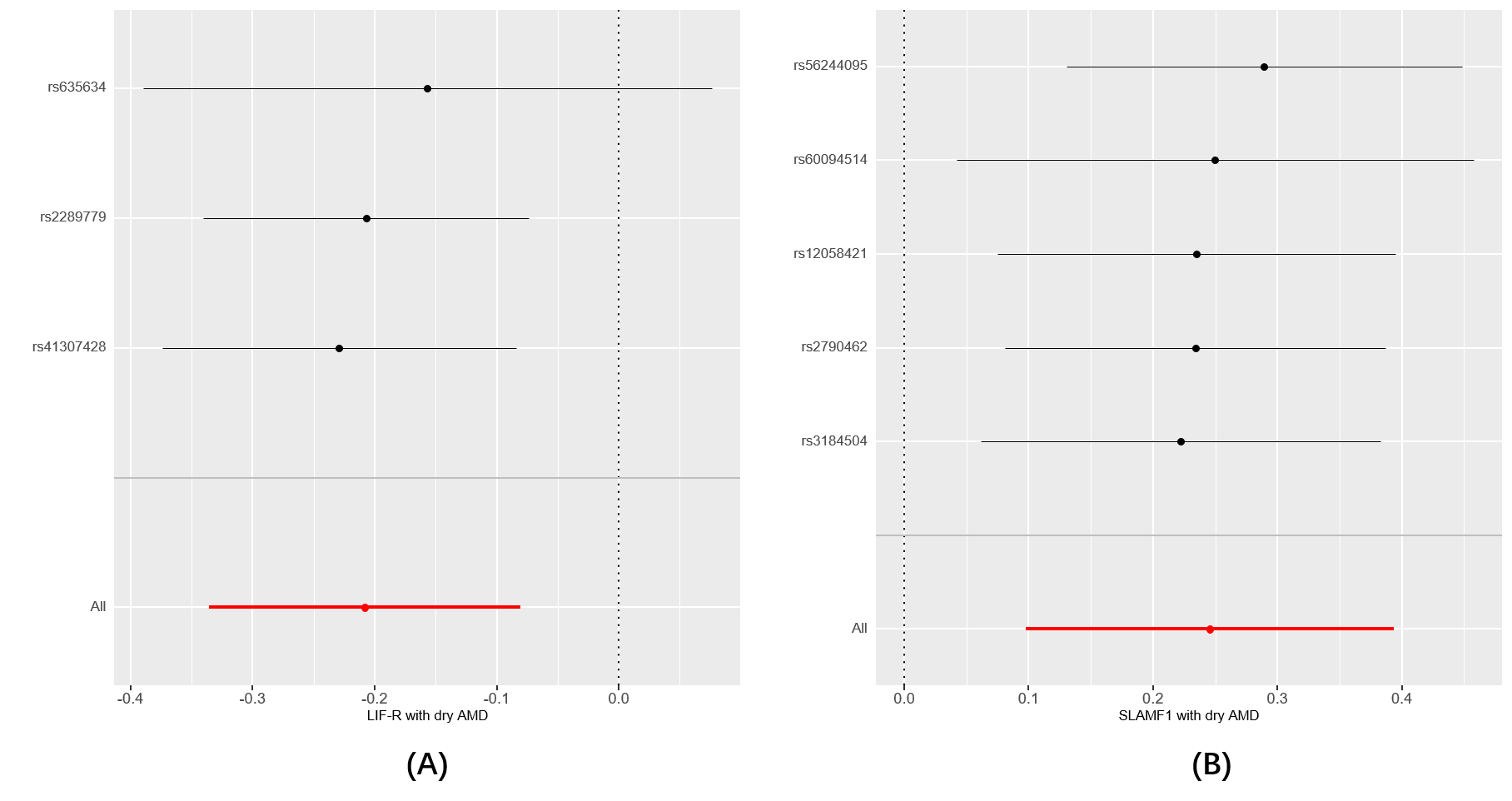


**Supplementary Figure 3**. MR leave-one-out sensitivity analysis of systemic inflammatory regulators-associated SNPs with risk of dry AMD. The genetic relationship between LIF-R, SLAMF1 and dry AMD, denoted as A to B, respectively. Abbreviations: LIF-R, Leukemia inhibitory factor receptor; SLAMF1, Signaling lymphocytic activation molecule.
